# The Proposed Registration of Nurses

**Published:** 1890-01-11

**Authors:** 


### The Proposed Registration of Nurses.

*(Continued from page 181.)*

Copies of this memorial may be obtained from, and further names may be sent to, Dr. Steele, superintendent, Guy's Hospital, London, S.E.

Some Provincial and Scotch Hospitals and Nurse-Training Schools Opposed to a Common Register, not Hitherto Published.

We, the undersigned, beg to submit the following statement, which we think it desirable to make, in view of a paragraph which has been published on the subject of the registration of nurses, in which we note with surprise the statement that the main object of the British Nurses' Association "is in conformity with a great public want, and a widespread professional demand."

We would wish to point out that those who represent the largest nursing interests in the metropolis and throughout the country, and who have the most to do with the training and examination of nurses, have not only declined to take part in the association, but consider that its proposed enrolment of nurses in a common register, if carried out, would (1) lower the position of the best trained nurses, (2) be detrimental to the advancement of the teaching of nursing, (3) be disadvantageous to the public, and (4) be injurious to the medical practitioner.

We hope that a final judgment upon this important matter will be postponed until the views of those who are opposed to the aims of this association have been expressed and examined. We further consider it our duty to state that if a charter be applied for on the lines stated in the prospectus of the British Nurses' Association, we shall feel it to be incumbent upon us to offer thereto all legitimate opposition in our power.

*Fourth List—Provincial Hospitals and Nursing Institutions.*

Birmingham: Queen's Hospital.—Mary Seymour Roberts, superintendent of nurses.

Cheltenham General Hospital.—C. E. Kroker King, treasurer; E. T. Wilson, physician; K. E. Tatham, matron.

Essex and Colchester Hospital.—Horace E. Green, chairman; Alexander Wallace, M.D., physician; Emma Appleton, matron.

Glamorganshire and Monmouthshire Infirmary, Cardiff. Franklen G. Evans, M.D., chairman; A. Sheen, M.D., hon. surgeon to infirmary and lecturer to nursing staff; Jno. Thomas, M.R.C.S., etc., house surgeon to Cardiff Infirmary and lecturer to nursing staff; A. M. Francis, matron and superintendent of nurses.

Gloucester County Hospital.—T. S. Ellis, consulting surgeon; Raynor W. Batten, senior physician; H. E. Waddy, senior surgeon; Oscar Clark, physician; E. Yeats, matron.

Leeds General Infirmary.—R. Benson Jowitt, treasurer and chairman; A. F. McGill, F.R.C.S., hon. surgeon; H. Littlewood, resident medical officer and instructor of nurses; L. M. Gordon, lady superintendent of nurses; E. Fisher, assistant superintendent of nurses.

Leeds Trained Nurses' Institution.—Frederick Baines, chairman; M. J. Dawson, superintendent of nurses.

Liverpool Northern Hospital.—E. H. Dickenson and James Barr, physicians; Chauncey Puzey and Damar Harrison, surgeons; J. B. Anderson, matron.

Liverpool Royal Infirmary.—H. B. Gilmour, chairman.

Liverpool Training School and Home for Nurses.—William Rathbone, president; Charles Langton, chairman of committee; Elizabeth Staines, superintendent of training school.

Liverpool Workhouse Hospital.—J. W. Cropper, chairman of nursing committee; R. Robertson and William Alexander, medical officers; Margaret Stewart, superintendent of nurses.

Liverpool Royal Southern Hospital.—George H. Horsfall, president; John Cameron, M.D., chairman of medical board; M. Gordon, matron.

Macclesfield General Hospital.—Thomas Lockett, chairman; H. M. Fernie, senior medical officer and lecturer to nursing staff; Sarah A. Yoke, lady superintendent of nurses.

Manchester Nursing Institution.—Louisa Potter, hon. secretary; M. Nicholls, lady superintendent.

Newcastle-on-Tyne: Nursing Home and Training School.—Fred Page, surgeon, Royal Infirmary; Esther Emery, matron.

Southport Infirmary.—J. C. Barrett, chairman; W. Walker, vice-chairman; Mary Lambert, matron.

Winchester: Royal Hants County Hospital.—R. Jones Bateman, chairman; B. N. Earle, M.D.; W. M. Harman, M.D., F.R.C.S.; T. C. Langdon, F.R.C.S., resident medical officer, and lecturer to and examiner of nurses; W. P. Terry, Major, secretary; E. Suckling, matron and superintendent of nurses.

Worcester Infirmary.—W. Strange, M.D., senior physician; Horace Swete, M.D., hon. physician, and Rory Fletcher, house surgeon, instructors of nurses; Jane Maclelland, matron; Emily Bartlett, assistant matron.

Worcester: St. John's House Nursing Institution.—Edmund A. H. Lechmere, secretary-general, Order of St. John of Jerusalem; John Gott, D.D., Dean of Worcester and chairman of committee; the Lord Bishop of Ely, and Florence Compton, members of committee; G. W. Crowe, M.D., and Tom Bates, M.R.C.S., members of the council of the Order of St. John; Janet Topping, lady superintendent of nurses.

*Some Scotch and Irish Hospitals and Institutions.*

Banff: Chalmer's Hospital.—Robert Duncan, treasurer; W. Ferguson, visiting surgeon; A. S. Ingram, lecturer to nurses; E. M. C. Gray, matron.

City of Dublin Hospital.—Susan Beresford, lady superintendent.

City of Dublin Hospital—Nursing Institution.—Gerald Fitzgibbon, Lord Justice of Appeal; J. D. Pratt, F.R.C.S., honorary secretary; E. L. Fitzgerald, M.D., and R. W. Harley, directors; Annie Fitzgerald, manager.

Edinburgh Royal Infirmary.—Thomas Annandale, regis professor of clinical surgery; John Duncan, lecturer on clinical surgery, senior ordinary surgeon; J. O. Affleck, M.D., lecturer on clinical medicine and ordinary physician; J. Halliday Croom, M.D., lecturer on diseases of women and ordinary physician; A. G. Miller, ordinary surgeon; John Chiene, professor of surgery; P. H. Maclaren, ordinary surgeon; Andrew Smart, M.D., senior assistant physician; Alexander James, M.D., assistant physician; Charles W. Cathcart, assistant surgeon; F. E. Spencer, superintendent of nurses; Annie Grant, assistant superintendent of nurses and teacher of probationers; C. H. Fasson, D.S.G., superintendent.

Glasgow Fever Hospital.—Amelia Sinclair, matron.

Glasgow Royal Infirmary.—Hugh Brown, chairman; M. Thomas, M.D., medical superintendent; Annie Eleanor Wood, matron.

Glasgow Western Infirmary.—William Kerr, chairman; James Finlayson, M.D., physician, formerly lecturer to nurses; Hugh Cameron, surgeon; John Alexander and David Newman, lecturers to nurses; A. W. Russell, M.D., medical superintendent; Elizabeth Clyde, matron.

